# (Re)form with Substance? Restructuring and governance in the Australian health system 2004/05

**DOI:** 10.1186/1743-8462-2-19

**Published:** 2005-08-24

**Authors:** Mark Rix, Alan Owen, Kathy Eagar

**Affiliations:** 1 Centre for Health Service Development, Faculty of Commerce, University of Wollongong, NSW, 2515, Australia

## Abstract

The Australian health system has been the subject of multiple reviews and reorganisations over the last twenty years or more. The year 2004–2005 was no different.

This paper reviews the reforms, (re)structures and governance arrangements in place at both the national and state/territory levels in the last year. At the national level some progress has been made in 2004/05 through the Australian Health Ministers' Council and there is now a national health reform agenda, albeit not a comprehensive one, endorsed by the Council of Australian Governments (COAG) in June 2005. Quality and safety was an increasing focus in 2004–2005 at both the national and jurisdictional levels, as was the need for workforce reform. Although renewed policy attention was given to the need to better integrate and coordinate health care, there is little evidence of any real progress this last year. More progress was made on a national approach to workforce reform.

At the jurisdictional level, the usual rounds of reviews and restructuring occurred in several jurisdictions and, in 2005, they are organisationally very different from each other. The structure and effectiveness of jurisdictional health authorities are now more important. All health authorities are being expected to drive an ambitious set of national and local reforms. At the same time, most have now blurred the boundary between policy and service delivery and are devoting significant resources to centrally 'crisis managing' their service systems. These same reasons led to decentralisation in previous restructuring cycles. While there were many changes in 2004–2005, and a new national report to COAG on health reform is expected at the end of 2005, based on current evidence there is little room for optimism about the prospects for real progress.

## Review

The Council of Australian Governments' (COAG) 15th meeting on 3 June 2005 in Canberra endorsed a national health reform agenda with an unusual level of national consensus. The heads of Governments agreed that Australia has one of the best health systems in the world, albeit with room for improvement, particularly in areas where governments' responsibilities intersect. After several years of apparent stalemate, it seemed that the discussion had re-opened on ways to improve Australia's health system. In its most ambitious section, the COAG 2005 Communique "agreed that where responsibilities between levels of government need to change, funding arrangements would be adjusted so that funds would follow function." [1]

The governments stated in their Communique that the health system can be improved by clarifying roles and responsibilities, and by reducing duplication and gaps in services. They recognised that many Australians, including the elderly and people with disabilities, still face problems at the interfaces of different parts of the health system. They restated the aim of integration and a smooth transition between acute care, home and residential care, and helping younger people with disabilities. The necessary themes of workforce supply and flexibility, prevention, electronic records, a national call centre, and rural and remote services were reinforced. Senior officials were given till December 2005 to come up with a plan of action to progress these reforms.

The core ideas behind this change of climate have been well rehearsed in both health bureaucracies and the submissions of industry groups. In 2004 the Australian Healthcare Association (AHA) released five policies, the first of which called for a *National Health System *so that, by 2008, all Australian governments will have adopted a nation-wide approach to health policy and service delivery. AHA argued that "a National Health System is fundamental to successful health system reform in Australia and will provide access to health care services for Australians irrespective of borders or payers" [2].

The rest of the policy agenda was a call for a *National Package of Healthcare Services*, so that the next Australian Healthcare Agreement (2008–2013) would govern all public sector health programs and services administered by all Australian governments in partnership [3]. This was accompanied by a *National Approach to Quality and Safety *in Health [4], and policies for *better integration and coordination of health care *[5], and a *national approach to workforce reform *[6].

While no doubt ambitious, there is little dispute about the merit of these policies and their potential impact on health in Australia. Given the renewed relevance of larger scale reform under the 2005 COAG announcements, these policy signposts form a useful framework by which to assess the state of the Australian health care system and its attempts at reform in 2004/05.

## The state of play in Australian-state/territory government relations in 2004/05 – ritual or reform?

Without a clear strategy to move to a more truly national system, all reform is going to be counterbalanced by the inbuilt tendencies of the current system to move towards increased fragmentation. In a recent overview and assessment of Australian health system restructuring, Dwyer lamented that 'Unfortunately, the Commonwealth: state responsibility split, the one structural barrier most central to the systemic weakness of Australian primary care (and therefore most important for the capacity to develop and support new models of care for chronic diseases), is one that a state can't address, at least not alone [7].'

Before the COAG announcement in June 2005, there was little prospect of progress. The Prime Minister had announced in October 2004 a Task Force headed by Andrew Podger, the previous head of the Department of Health and Ageing (DHA). Consisting of officers from the Departments of Prime Minister and Cabinet, Health and Ageing and the Treasury, its role was to review the operation of health policy to examine how to improve the delivery of health services. Finalised in early 2005, its report has not been released. Federal Health Minister Tony Abbott referred briefly to it earlier in 2005 by commenting that it has been commissioned so that "the Government can respond to any state proposal" [8].

At that time Minister Abbott had outlined the problems of the health system from the point of view of the Australian Government when he addressed the Committee for Economic Development of Australia Conference in February 2005. In his address, Minister Abbott was not concerned with primary care, but with the 'big health issue' for 2005 – hospitals. For the Minister, this was primarily a matter for the states and territories rather than the Commonwealth, and a good opportunity for some "free-kicks". He pointed out that Section 51 of the Constitution relegates the Commonwealth to little more than a funding authority having no operational control of public hospital systems in the subordinate jurisdictions. While he did concede that it would make more sense for one level of government to be responsible for the entire health system, the real issue of the day was not so much who funds hospitals but how they are managed.

The Minister complained that 'years of poor management mean that public hospital patients now face long waits for essential as well as elective treatment (Abbott 2005).' Private hospitals, in stark contrast, were in the business of providing patients 'with what they want, when they want it'. The challenge for the federal government was to exercise effective leadership over the public hospital systems that are run by the states and the territories and private sector hospitals which, the minister remarked, 'aren't run by the government at all [8].'

Fortunately, the national health reform agenda is too important to be left to health ministers alone. Continuing its previous calls for more reform, the Productivity Commission recommended in February 2005 an independent public review of Australia's health care system, as the first step in the development of an integrated reform program. "The review should include consideration of: the future determinants of demand for and supply of health services; health financing (including Federal/State responsibilities and their implications); coordination of individual services (including with aged care); the interface between public and private services; information management; and the appropriate balance of resourcing between prevention and treatment" [9].

If a revived impetus for a national approach to reform through heads of government can broaden the debate beyond who runs hospitals, and look at primary care and the relationships to residential aged care and community care, then the latest review under COAG will be able to build on progress that has been made under the auspices of the health ministers' conferences at national level over the last year.

## Toward a national reform agenda – small steps in the right direction?

The perennial issue of the Commonwealth: state/territory split of responsibility for health is hardly the only matter of concern in assessing the effectiveness of public health policy in Australia – even though, as Dwyer comments, it is 'probably the single most significant problem in health system design [[7], p 4].' The treatment and prevention of chronic disease are also of great concern with chronic disease accounting for '80% of the total burden of disease' and approximately '40% of total health expenditure' [[7], p 6].

Chronic disease and related issues were high on the agenda of the meeting between Australian Health Ministers and clinicians in Hobart in July 2004. The meeting was reviewing the progress of the *Health Reform Agenda *agreed to at the November 2003 meeting of Health Ministers (the Health Ministers had first agreed to the Reform agenda at their April 2003 meeting). Instead of a large systematic process of reform as suggested by the Productivity Commission and others, health reform in Australia is to be progressed in a series of small steps.

The first step was the establishment of a Health Reform Agenda Working Group (HRAWG) to progress the Health Reform Agenda. It reports to the Australian Health Ministers' Advisory Council (AHMAC). As shown in Table 1, there are optimistic signs in 2005 of some progress toward genuine reform, albeit in small steps.

**Table 1:** Outcomes of Australian Health Ministers' Conferences 2004–2005 in relation to structural reform

**Agreement / Outcome**	**Press release date**
Agreement to take "immediate action to progress reform of the Australian health care system in the areas of after hours GP services; aged care; chronic disease and cancer services; medical workforce planning; and, renal disease services" [10]	28 November 2003
Establishment of a national nursing taskforce to drive major nursing education and workforce reforms [11]	28 November 2003
Release of Australia's first national health workforce strategic framework [12]	23 April 2004
Agreement to take further steps "to progress reform of the Australian health care system in the areas of after hours GP services; aged care; chronic disease and cancer services; medical workforce planning; and, renal disease services" [13]	23 April 2004
Agreement on a nationally consistent approach to medical registration [14]	23 April 2004
Agreement on the first National Health Workforce Action Plan [15]	29 July 2004
Agreement to continue the Health Reform Agenda and the future priorities for reform [16]	29 July 2004
Agreement to establish a Review of the Future Governance Arrangements for Safety and Quality in Health Care [17]	29 July 2004
Agreement to establish a new national entity to drive critical e-health initiatives – NEHTA [18]	28 January 2005
Endorse development of a National Framework for Action on Dementia [19]	28 January 2005

At the April 2004 meeting, the Health Ministers acknowledged the need for immediate action to ensure progress in reforming after hours GP services, aged care, chronic disease and cancer services, medical workforce planning, and renal disease services. Among other matters, the Health Ministers agreed to establish a '*set of principles*' that would allow jurisdictions to work together towards improving delivery of after hours GP services in certain regions and building collaborative working relationships with emergency departments in public hospitals.

They also agreed to a range of initiatives such as enhanced transition care, rehabilitation and step-down care that would improve the transition between acute and aged care services. In addition, the Ministers reached agreement to finalise an integrated Chronic Disease Strategy and Service Improvement Framework for Cancer services [13].

At their July 2004 meeting, Health Ministers were asked by clinicians to consider three issues that they regarded as important:

• integration and coordination of services at the community-based and hospital-based services interface;

• improving the community's access to better health outcomes, in particular, for children, people with chronic care needs, older Australians, and indigenous Australians; and

• the need for a sustainable, skilled and flexible workforce to enable the adequate provision of health services into the future.

As these issues were already on their Health Reform Agenda, Health Ministers agreed to endorse child health and well being as a specific area for reform. In turn, the clinicians recommended that a way to progress a number of the important items on the Reform agenda was to conduct a trial in each state and territory of specific services that integrate community-based and hospital based-services, suggesting coordinated chronic care and integrated aged care as possible cases [16]. Those trials are yet to eventuate.

At the same meeting, the ministers agreed to establish a Review of the Future Governance Arrangements for Safety and Quality in Health Care. The review is to advise on future arrangements for the effective leadership and national coordination of safety and quality initiatives in health care. It is to report before the Australian Council for Safety and Quality in Health Care completes its current term in June 2006. National governance arrangements for leadership and coordination of safety and quality in health care were accordingly included in the Terms of Reference of the health care safety and quality governance arrangements review [17]. In parallel, several states and territories introduced their own initiatives, reflecting the increasing priority being given to governance arrangements for quality and safety in 2004–2005.

Meeting in Sydney in January 2005, the Australian Health Ministers' Conference agreed to establish the National E-Health Transition Authority (NEHTA). This is a new entity in the form of a company limited by guarantee and governed by a board of directors comprising the CEOs of the national, state and territory Health Departments. Its core activities include 'the development of timelines for the urgent advancement of the e-health agenda; option assessment and business case development; standards development and implementation support; and provision of advice and resources to assist implementation of already agreed solutions [18].' It is also expected to advance other significant national priorities in key areas including clinical data standards and terminologies, consent models, electronic health record (EHR) standards, and health informatics industry reform.

## Incremental and crisis-driven reforms at state/territory level – just more change or so me real progress?

Australian states and territories have a long history of independent reviews leading, cyclically, to the centralisation and decentralisation of management and governance at various times. In 2004–05, Australian jurisdictions are, in the main, in a centralisation phase. Queensland is the subject of an independent review at the time of writing while Western Australia has a Health Reform Implementation Taskforce in progress. Dwyer [7] reviewed the round of reviews in the Australian health system between 2002 and 2004. These resulted in restructuring in New South Wales, South Australia, the Northern Territory, Western Australia, and the ACT. There is a strong tendency towards increasing centralisation so that, in 2005, 6 of 8 jurisdictions now directly manage public sector health services, with Victoria and South Australia having mixed models. With the recent centralisation of management in New South Wales, Dwyer calculated that two thirds of Australians now live in areas under centralised control [7].

Given this, the structure and effectiveness of jurisdictional health authorities is becoming increasingly important in determining whether reforms are achieved in areas such as clinical governance, quality and safety and others included in the National Reform Agenda. This is especially the case given that, at the same time, the centralised authorities will continue to devote considerable resources to responding to each new 'crisis' in their service system.

Table 2 summarises the management structures in place in each jurisdiction in May 2005. As this summary indicates, there are significant differences in the role and scope of the various health authorities. This has important implications in relation to structural opportunities to reform and improve the coordination and planning of service delivery, particularly for those with complex and continuing care needs. At the same time, there is little similarity in how the various departments are organised, as reflected in their executive level divisional structures (listed in alphabetical order in the table).

**Table 2:** Management of health and human services by jurisdiction – the state of play in 2004–2005

**Jurisdiction**	**Scope**	**Organisational divisions**	**Regions**
**Australian Government**	Health and Ageing Separate authorities for Family and Community Services and Veterans' Affairs	Acute Care Ageing and Aged Care Business Health Services Improvement Medical and Pharmaceutical Services Office of Aboriginal and Torres Strait Islander Health Population Health Portfolio Strategies Primary Care	Each states and territory is a region
**ACT**	Health Separate authorities for Disability, Housing and Community Services. ACT Emergency Services Authority provides the ambulance service Separate Community and Health Services Complaints Commissioner established in late 2004	Allied Health Adviser Clinical operations Financial and Risk Management Government Relations and Planning Human Resource Management Information Services Nursing and Midwifery Office Policy Population health	None. All services directly managed by the Department
**Northern Territory**	Health and community services Separate authorities for Community Development, Sport and Cultural Affairs Separate Health and Community Services Complaints Commission	Aboriginal Health, Family & Social Policy Acute Care Community Services Corporate Management Services Health Services Information Strategy & Quality	None. All services directly managed by the Department St John Ambulance Service is separately incorporated, as are some Aboriginal Health Services
**NSW**	Health Separate authorities for Ageing, Disability and Home Care, Housing, Community Services and Medical Research Separate Health Care Complaints Commission	Health System Performance Health System Support Population Health Strategic Development	8 Area Health Services (no Boards) reporting directly to the department plus: Ambulance Service of NSW Children's Hospital at Westmead (with Board of Directors) Justice Health Clinical Excellence Commission NSW Cancer Institute
**Queensland**	Health Separate authorities for Child Safety, Communities, Emergency Services [including Ambulance Service], Housing, Disability Services Separate Health Rights Commission of Queensland	Health Services Information Innovation and Workforce Reform Resource Management. Strategic Policy and Government Liaison	3 Zones 37 Districts within zones All services directly managed by the Department
**South Australia**	Health Department of Families and Communities manages other human services, including Aged and Community Care Separate Health and Community Services Complaints Commission announced in 2004 Separately incorporated bodies deliver ambulance services Veterans Repatriation Hospital separate Hospitals and Dom care separate incorporation	Population and Environmental Healthy SA Service Planning State Dental managed in a region Mental Health managed in a region Drug and Alcohol managed in a region SA Health Reform	2 metropolitan health regions and Children, Youth and Women's Health Service with own Boards. 4 country regional health services
**Tasmania**	Health and Human Services Separate Health Complaints Commissioner	Children and Families Community, Population and Rural Health Corporate Services Hospitals and Ambulance Housing Tasmania	None. All services directly managed by the Department
**Victoria**	Health and Human Services Separate Office of the Health Services Commissioner Office for Children that now reports to Minister for Children	Disability Services Financial & Corporate Services Housing & Community Building Metropolitan Health & Aged Care Services Operations Policy & Strategic Projects Rural & Regional Health & Aged Care Services	8 departmental Regions 12 Melbourne networks with own boards within metropolitan regions 71 agencies with own boards in rural regions Victorian Ambulance Service
**Western Australia**	Health Separate authorities for Community Development, Disability Services and Housing, Office of Health Review. Separate Office of Safety and Quality	Clinical Policy Division. Statewide Health Support Population Health Division Country Health Services Central Wait List Bureau	3 Area Health Services, 1 Country Health Service and Women's and Children's Health Service, all directly managed by the Department St John Ambulance Service is separately incorporated

At a national level, the department is responsible for both health and ageing. But the health care needs of war veterans are the responsibility of the Department of Veterans Affairs (DVA) and not the Department of Health and Ageing (DHA). The 'ageing and aged care' functions of DHA include community care programs and services such as the Home and Community Care (HACC) program that are managed by the health authority in all but two jurisdictions. New South Wales has a separate Department of Ageing, Disability and Home Care. In South Australia, the previous Department of Human Services was split on 1 July 2004 into two, with a new Department of Families and Communities taking responsibility for, among other portfolios, community care and disability.

In 2005, an authority with broader human and community services responsibilities is managing health care in the Northern Territory, Tasmania and Victoria. These other responsibilities include, among some others, disability services and housing. Neither function is now within scope of the health departments in the other jurisdictions.

Tasmania has the broadest role and is responsible for both the policy and direct operations of its ambulance service. This is not the case in either Victoria or the Northern Territory where the department manages policy but ambulance services are separately incorporated. In other states with a narrower 'health department', ambulance services are managed by departments of emergency services (ACT, Queensland), by the health department (New South Wales) or are separately incorporated services (South Australia).

All jurisdictions now have independent authorities (however named) to review health care complaints. The ACT and South Australia established theirs in 2004. However, important differences in the philosophy and role of such bodies were identified in evidence given to the Special Commission of Inquiry into Campbelltown and Camden Hospitals [20], particularly in relation to their role in 'blaming' those responsible for errors.

Western Australia and New South Wales have gone further. An independent Office for Safety and Quality in Health Care was established in Western Australia in 2002. It is responsible for supporting the establishment of effective quality and safety systems, as well as investigating complaints. New South Wales established a separate Clinical Excellence Commission in 2005 (replacing its previous Institute of Clinical Excellence). Not surprisingly, both initiatives followed major media coverage of 'hospital scandals' in those two states.

Organisational and executive structures differ between jurisdictions. As one example, public (or population) health is its own division, and reports directly to the departmental head, in the ACT, WA and NSW. In Victoria, it is an office within the Rural and Regional Health and Aged Care Services Division while in Queensland it is a branch within the Health Services Division. Population health functions in the Northern Territory also sit in a Health Services Division, but not in one branch. Instead, population health is the responsibility of several sections including the Centre for Disease Control and a Health Development and Oral Health Branch. In Tasmania, population health is a subdivision of the Community, Population and Rural Health Division. At least in part, these differences reflect the scope of the various departments. However, there is no evidence to suggest whether any of these structures produce more effective policy than others. Nor is there evidence on what structure is best able to manage the health system and its reform.

As one further example, workforce reform (one of the five 2004 AHA policies and also on the Australian Health Ministers Health Reform Agenda) is managed differently across the jurisdictions. In 2005, Queensland has a new Innovation and Workforce Reform Directorate while Western Australia announced in May 2005 the creation of a new Clinical Reform and Policy Division. In other jurisdictions, there is either no organisational unit responsible for workforce reform or it is incorporated in the functions of other sections such as human resource departments. As before, there is no evidence to suggest whether any of these structures will be more effective than others in delivering on the workforce reform agenda.

One reason for the differences between jurisdictions appears to be the circumstances that triggered each of their latest restructures. As Dwyer [7] notes, all but one (NSW) arose from an independent review with the reorganisation of NSW coming in the aftermath of a hospital 'scandal' that attracted much media coverage. On the same basis, a number of reviews are now underway in Queensland.

In response to the so-called 'Doctor Death' scandal in relation to the appointment of Dr Jayant Patel in Bundaberg (Queensland), the Queensland Premier (not the Health Minister) announced in April 2005 the Queensland Health Systems Review. Its establishment had been the suggestion of the major doctors lobby group, the Australian Medical Association (AMA) [21]. This major review of Queensland Health's administration, management and performance systems is due for public release on 30 September 2005.

At the same time, three other inquiries have been commissioned. A Commission of Inquiry has been established to investigate events at Bundaberg Hospital, including the role and conduct of the Queensland Medical Board in relation to overseas trained medical practitioners. Like the Queensland Health Systems Review, it has also been asked to consider changes to recruitment, employment and supervision of medical practitioners, management of complaints and measures to increase the availability of medical practitioners across the State. In parallel, the Crime and Misconduct Commission is also conducting a public inquiry into Queensland Health's handling of complaints regarding care at Bundaberg Hospital and a Queensland Health review of clinical services at Bundaberg Hospital is also underway [22].

The Queensland Health Systems Review has broad terms. The administrative systems to be examine include (among other matters) district and corporate organisational structures and layers of decision making; corporate planning and budgeting systems; the effectiveness of performance reporting and monitoring systems; quality and safety systems; and clinical audit and governance systems. On the workforce front, it will examine recruitment; retention; training and clinical leadership. It will also review performance management systems including asset management and planning, information management and monitoring systems.

Regardless of the detail, it seems unlikely that the status quo will remain in Queensland in 2006. No doubt other jurisdictions will be watching in an attempt to learn the lessons.

## Conclusion

We noted that the five AHA policies released in 2004 form a useful framework by which to assess the state of the Australian health care system and its attempts at reform in 2004/05. In 2005, Australia does not have a *National Health System*. Some progress has been made in 2004/05 and there is now a national health reform agenda under COAG. However, the current evidence suggests it is still a reform agenda in separate bits and will not be systemic. Little or no progress was made toward a *National Package of Healthcare Services *and there are no indications of any progress in the near future on that front. A *National Approach to Quality and Safety *is emerging, with significant advances in some jurisdictions. *Better integration and coordination of health care *remained a fashionable idea in 2004/05 but this goal has been acknowledged as important for decades and real progress is dependent on more systemic change. More progress was made on a national approach to *workforce reform *with the release of a national "strategic framework to guide national health workforce policy and planning throughout the decade". But a framework is still a long way from a strategy.

At the state and territory level, reviews and restructuring continued in several jurisdictions. In 2005, there are significant organisational differences between them, with little evidence of the strengths and weaknesses of the different approaches. What is becoming increasingly apparent is that the structure and effectiveness of jurisdictional health authorities is now more important. All health authorities are being expected to drive an ambitious set of national and local reforms. At the same time, most have now blurred the boundary between policy and service delivery and are devoting significant resources to 'crisis managing' their service systems. These same reasons led to decentralisation in previous restructuring cycles.

With scandals, public criticism and concern with rising costs increasingly being the impetus to restructure, the prospects for 2006 are for more of the same. At the same time, delivering on the reform promises of 2004/05 will become increasingly difficult but more important than ever.

## Competing interests

The author(s) declare that they have no competing interests.

## References

[B1] Council of Australian Governments 2005 Communique, June 3. http://www.coag.gov.au/meetings/030605/index.htm#health.

[B2] Australian Healthcare Association AHA 2004 Policy 1. A National Health System. http://www.aushealthcare.com.au/publications/publication_details.asp?pid = 90.

[B3] Australian Healthcare Association AHA 2004 Policy 2. A National Package of Healthcare Services. http://www.aushealthcare.com.au/publications/publication_details.asp?pid = 91.

[B4] Australian Healthcare Association AHA 2004 Policy 3. A National Approach to Quality and Safety in Health. http://www.aushealthcare.com.au/publications/publication_details.asp?pid = 92.

[B5] Australian Healthcare Association AHA 2004 Policy 4. Better integration and coordination of healthcare. http://www.aushealthcare.com.au/publications/publication_details.asp?pid = 93.

[B6] Australian Healthcare Association AHA 2004 Policy 5. A National Approach to Workforce Reform. http://www.aushealthcare.com.au/publications/publication_details.asp?pid = 94.

[B7] Dwyer JM (2004). Australian health system restructuring – what problem is being solved?. Australia and New Zealand Health Policy.

[B8] Abbott T 2005 Minister for Health and Ageing The Hon Tony Abbott MHR, addresses the Committee for Economic Development of Australia conference in Sydney. http://www.health.gov.au/internet/wcms/publishing.nsf/Content/health-mediarel-yr2005-ta-abbsp250205.htm.

[B9] Productivity Commission (2005). Review of National Competition Policy Reforms, Report no. 33. Canberra.

[B10] Australian Health Ministers' Conference 2003 Joint Communique Australian Health Ministers agree to Reform Agenda. http://www.health.gov.au/internet/wcms/publishing.nsf/Content/health-mediarel-yr2003-jointcom-jc001.htm.

[B11] Australian Health Ministers' Conference 2003 Joint Communique National Nursing and Nursing Education Taskforce. http://www.health.gov.au/internet/wcms/publishing.nsf/Content/health-mediarel-yr2003-jointcom-jc004.htm.

[B12] Australian Health Ministers' Conference 2004 Joint Communique National health workforce strategic framework. http://www.health.gov.au/internet/wcms/publishing.nsf/Content/health-mediarel-yr2004-jointcom-jc004.htm.

[B13] Australian Health Ministers' Conference 2004 Joint Communique Health Ministers Agree to Reform Agenda. http://www.health.gov.au/internet/wcms/publishing.nsf/Content/health-mediarel-yr2004-jointcom-jc001.htm.

[B14] Australian Health Ministers' Conference 2004 Joint Communique Australian Health Ministers agree on nationally consistent approach to medical registration. http://www.health.gov.au/internet/wcms/publishing.nsf/Content/health-mediarel-yr2004-jointcom-jc003.htm.

[B15] Australian Health Ministers' Conference 2004 Joint Communique National health workforce strategic framework. http://www.health.gov.au/internet/wcms/publishing.nsf/Content/health-mediarel-yr2004-jointcom-jc004.htm.

[B16] Australian Health Ministers' Conference 2004 Joint Communique Health Ministers Agree to continue reform agenda. http://www.health.gov.au/internet/wcms/publishing.nsf/Content/health-mediarel-yr2004-jointcom-jc006.htm.

[B17] Australian Health Ministers' Conference 2004 Joint Communique Review of Future Governance Arrangements for Safety and Quality in Health Care. http://www.health.gov.au/internet/wcms/publishing.nsf/Content/health-sqreview.htm.

[B18] Australian Health Ministers' Conference 2005 Joint Communique National entity to drive E-Health. http://www.health.gov.au/internet/wcms/publishing.nsf/Content/health-mediarel-yr2005-jointcom-jc001.htm.

[B19] Australian Health Ministers' Conference 2004 Joint Communique Australian Health Ministers endorse development of a National Framework for Action on Dementia. http://www.health.gov.au/internet/wcms/publishing.nsf/Content/Media+Releases+Communiques-1.

[B20] Walker B (2004). Final Report of the Special Commission of Inquiry into Campbelltown and Camden Hospitals. http://www.lawlink.nsw.gov.au/special_commission.

[B21] Beattie P 2nd Inquiry Will Check Health Systems To Aim For Better Results. http://statements.cabinet.qld.gov.au/cgi-bin/display-statement.pl?id = 6420&db = media.

[B22] Queensland Health (2005). Other reviews. http://www.healthreview.com.au/other_reviews.asp.

